# Ninety-day readmission and early complications after coding-defined cementless-category versus cemented-category primary total knee arthroplasty

**DOI:** 10.1186/s42836-026-00418-2

**Published:** 2026-08-05

**Authors:** David Maman, Yaniv Steinfeld, Yaron Berkovich

**Affiliations:** 1 Faculty of Medicine, Technion Israel Institute of Technology, Haifa, 2611001 Israel; 2 Carmel Medical Center, Haifa, 3436212 Israel

**Keywords:** Total knee arthroplasty, Readmission, Administrative database, Nationwide readmissions database, Propensity score matching, ICD-10-PCS, Cemented fixation, Cementless fixation

## Abstract

**Background:**

Use of cementless total knee arthroplasty (TKA) has increased in recent years, but contemporary short-term administrative comparisons with cemented fixation remain limited. This study compared 90-day readmission and early postoperative complications after ICD-10-PCS coding-defined cementless-category versus cemented-category primary TKA using a large U.S. administrative readmission database.

**Methods:**

The 2020–2022 Healthcare Cost and Utilization Project Nationwide Readmissions Database (NRD) was queried for adults undergoing elective unilateral primary TKA for osteoarthritis. Only cases performed on hospital day 0 using common cemented and cementless ICD-10-PCS procedure codes were included. Fixation category was defined using ICD-10-PCS administrative procedure coding and was analyzed as coding-defined cementless-category versus cemented-category primary TKA. Non-elective admissions, bilateral procedures, revision or reoperation cases, fracture-, malignancy-, infection-, and COVID-19-related admissions, and index discharges after September were excluded. Readmissions involving contralateral knee arthroplasty were also excluded. Propensity-score matching was performed on the unweighted analytic sample, and DISCWT was applied after matching to generate weighted national estimates and proportions. The primary outcome was 90-day all-cause readmission. Secondary outcomes included intraoperative fracture, blood loss anemia, blood transfusion, stratified in-hospital complications, readmission burden, grouped primary causes of readmission, and grouped principal procedures performed during readmission.

**Results:**

The 1:1 matched analytic cohort included 23,140 unweighted admissions in each coding-defined fixation category. After applying DISCWT, these corresponded to weighted national estimates of 40,490 cemented-category and 40,501 cementless-category primary TKA admissions. Coding-defined cementless-category TKA was associated with a lower weighted 90-day readmission proportion compared with cemented-category TKA (5.17% vs 5.76%; OR 0.89, 95% CI 0.84–0.95; *p* < 0.001), as well as lower weighted proportions of blood loss anemia (11.28% vs 13.13%; OR 0.84, 95% CI 0.81–0.88; *p* < 0.001) and any in-hospital complication (13.55% vs 15.36%; OR 0.86, 95% CI 0.83–0.90; *p* < 0.001). Intraoperative fracture was more frequent in the cementless-category group (0.30% vs 0.18%; OR 1.67, 95% CI 1.25–2.23; *p* = 0.001). Blood transfusion rates were comparable between cohorts. Among readmitted patients, grouped primary readmission diagnoses and grouped principal procedures were heterogeneous and included infectious, wound-related, thromboembolic, cardiopulmonary, gastrointestinal, renal/urinary, mechanical/fracture-related, and other medical categories. Knee revision-related procedures represented only a minority of readmission procedures. In the revised age-centered interaction analysis, the fixation-category association at age 70 years was not statistically significant, while the fixation-category by age interaction remained significant.

**Conclusions:**

In this U.S. nationwide propensity-matched administrative analysis of elective unilateral primary TKA, coding-defined cementless-category and cemented-category admissions demonstrated modest differences in selected early complications and 90-day readmission patterns. These findings characterize short-term administrative outcomes and should be interpreted in the context of coding-based fixation classification and the absence of implant-level, radiographic, functional, and long-term survivorship data.

**Levels of Evidence:**

**Supplementary Information:**

The online version contains supplementary material available at 10.1186/s42836-026-00418-2.

## Introduction

Total knee arthroplasty (TKA) is one of the most commonly performed orthopaedic procedures worldwide, and its utilization is expected to continue rising substantially as the population ages and demand for arthroplasty expands [1]. While cemented fixation has historically remained the standard approach in primary TKA, interest in cementless fixation has increased in recent years [2–5]. This renewed attention has been driven by advances in implant design, porous surface technologies, and evolving patient-selection strategies, particularly in younger or higher-demand patients [3–5].

Despite this trend, the relative safety profile and short-term outcomes of cementless compared with cemented TKA remain incompletely defined [2–10]. Contemporary studies have reported mixed findings. Some investigations have reported shorter operative time and comparable early clinical outcomes with cementless fixation in selected cohorts [6, 8, 11]. In contrast, other database and registry studies have raised concerns regarding early revision, periprosthetic fracture, or infection-related complications in selected populations [7, 9, 10]. Importantly, much of the available literature has been derived from single-center studies, registry analyses, or retrospective databases, each with inherent limitations and variable ability to account for baseline differences between patients undergoing cemented versus cementless fixation [2, 4, 7, 9, 10].

Early postoperative outcomes, including in-hospital complications and short-term revision or readmission-related events, have become increasingly important quality metrics in contemporary arthroplasty care [7, 9–11]. Clarifying whether fixation strategy influences these early outcomes is clinically relevant as cementless TKA is adopted more broadly, including in more diverse and potentially older patient populations [4, 5, 7].

Using a large, nationally representative administrative readmission database, the present study aimed to compare ICD-10-PCS coding-defined cemented-category and cementless-category primary TKA admissions with respect to in-hospital complications and 90-day readmission outcomes. To reduce measured baseline differences, we applied propensity-score matching to create closely comparable cohorts. We hypothesized that coding-defined fixation categories would demonstrate different short-term administrative perioperative risk profiles, particularly with respect to complications and readmission patterns.

## Materials and methods

### Data source

We conducted a retrospective cohort study using the Healthcare Cost and Utilization Project (HCUP) NRD for the years 2020 through 2022. The NRD is a nationally representative, all-payer inpatient database that captures discharge-level data from participating U.S. states and allows longitudinal tracking of patients within a single calendar year using validated linkage identifiers. This enables evaluation of readmission outcomes following an index hospitalization.

### Study population

Adult patients aged 18 years or older who underwent elective primary TKA were identified using ICD-10-PCS procedure codes recorded in the primary procedure field (I10_PR1). To ensure procedural consistency and minimize coding heterogeneity, the analysis was restricted to the following commonly utilized primary TKA codes:

- 0SRC0J9
- 0SRD0J9
- 0SRC0JA
- 0SRD0JA

A detailed code-to-category mapping table for these ICD-10-PCS procedure codes is provided in Supplementary Table S7. Other primary knee arthroplasty codes were excluded if they lacked a cemented or uncemented qualifier, represented partial or compartment-specific knee arthroplasty constructs, involved non-synthetic or unspecified device categories, or could not be assigned reproducibly to one of the two mutually exclusive coding-defined fixation categories.

To further standardize the cohort, only procedures performed for osteoarthritis were included, and surgeries were required to occur on hospital day 0, ensuring that index admissions represented planned primary arthroplasty procedures.

Exclusion criteria included:

- non-elective admissions,
- patients younger than 18 years,
- bilateral procedures,
- revision or reoperation procedures at the index admission,
- admissions associated with fracture, malignancy, or active infection,
- documented COVID-19 infection (ICD-10-CM U07.1),
- missing key demographic or hospital-level variables.

Because the NRD does not allow patient tracking across calendar years, only index admissions from January through September of each study year were included to ensure a complete 90-day readmission window.

### Exposure definition

The exposure of interest was fixation category as defined by ICD-10-PCS administrative procedure coding at the index hospitalization. Admissions were categorized as coding-defined cemented-category or cementless-category primary TKA based on the primary ICD-10-PCS procedure code recorded for the index arthroplasty. Because ICD-10-PCS administrative procedure codes do not provide implant-level detail, component-level fixation information, implant manufacturer data, cementing techniques, operative reports, or radiographic findings, these categories should not be interpreted as confirmed fully cemented or fully cementless implant constructs. This approach also cannot reliably identify hybrid fixation patterns or confirm osseointegration, radiographic fixation, durability, or long-term survivorship. Therefore, the exposure should be interpreted strictly as coding-defined cemented-category versus cementless-category primary TKA.

Accordingly, Supplementary Table S7 provides the explicit ICD-10-PCS code-to-category mapping used to define the coding-defined cemented-category and cementless-category primary TKA cohorts.

### Outcomes

The primary outcome was all-cause hospital readmission within 90 days of discharge following the index TKA.

Secondary outcomes included index-admission complications and readmission-related outcomes. Index-admission complications included intraoperative fracture, blood loss anemia, blood transfusion, and a composite measure of any postoperative in-hospital complication. The composite complication endpoint was additionally stratified into its individual coded components to improve clinical interpretability.

Readmission-related outcomes included time to readmission, readmission length of stay, readmission total hospital charges, primary diagnosis at readmission, and principal procedure performed during readmission. To better characterize the clinical context of readmissions, the primary ICD-10-CM diagnosis code of the first 90-day readmission was used to identify the leading specific causes of readmission in each fixation cohort. These diagnoses were summarized both as individual ICD-10-CM codes and as clinically grouped categories, including prosthesis-related infection, wound complications, cellulitis or soft-tissue infection, sepsis or bacteremia, thromboembolic events, cardiopulmonary diagnoses, gastrointestinal diagnoses, renal or urinary diagnoses, mechanical or fracture-related diagnoses, neurologic or cerebrovascular diagnoses, postoperative pain or hematoma, anemia or blood-loss diagnoses, syncope or hypotension, and other or mixed diagnoses. For grouped readmission diagnosis summaries, unweighted counts and DISCWT-weighted proportions among 90-day readmissions were reported.

Principal ICD-10-PCS procedure codes recorded during readmission were also examined to determine whether readmissions were associated with knee-specific procedures, revision-related or implant-removal procedures, soft-tissue debridement, fracture fixation, transfusion, gastrointestinal procedures, vascular access, urologic procedures, cardiopulmonary procedures, or other non-knee interventions. Because the NRD does not provide operative reports, implant records, laterality confirmation relative to the index procedure, or component-level revision details, procedure-code analyses were considered exploratory and were interpreted as reoperation-like or revision-related only when clinically consistent with the coded procedure.

To ensure that readmissions reflected events following the index procedure rather than staged contralateral arthroplasty, readmissions involving contralateral knee arthroplasty were excluded. In patients with multiple eligible readmissions, only the first readmission within 90 days was included in the analysis.

### Covariates

Covariates included demographic, clinical, socioeconomic, and procedural variables available within the NRD. Demographic and socioeconomic variables included age, sex, primary payer, and median household income quartile. Clinical comorbidities were identified using ICD-10-CM diagnosis codes and included hypertension, type 2 diabetes mellitus, obesity, chronic lung disease, chronic kidney disease, congestive heart failure, dyslipidemia, chronic anemia, osteoporosis, thyroid disorders, alcohol abuse, and obstructive sleep apnea. Use of robotic-assisted surgery was also recorded.

### Statistical analysis

To account for baseline differences between groups, 1:1 propensity-score matching was performed on the unweighted analytic sample using a nearest-neighbor algorithm without replacement and a caliper width of 0.1. The propensity model incorporated demographic characteristics, socioeconomic variables, hospital characteristics, procedural variables, and measured comorbidities. Matching was performed before applying NRD discharge weights. After matching, DISCWT was applied to generate weighted national estimates and weighted proportions. Therefore, unweighted analytic counts and DISCWT-weighted estimates are reported separately throughout the manuscript and supplementary tables.

Covariate balance before and after propensity-score matching was assessed using standardized mean differences (SMDs) for all variables included in the propensity-score model. Absolute SMD values below 0.10 were considered indicative of acceptable covariate balance. Pre-matching and post-matching SMDs are reported in Supplementary Table S1.

Categorical variables were compared using the chi-square test, and continuous variables were compared using the Student t-test or Wilcoxon rank-sum test, as appropriate. Within the matched cohort, multivariable logistic regression models were used to evaluate the association between coding-defined fixation category and outcomes, with results reported as odds ratios (ORs) and 95% confidence intervals (CIs).

For the age-interaction analysis, age was centered at 70 years using the variable AGE_C70 = age − 70. The interaction model included fixation category, centered age, the fixation-category × centered-age interaction term, and the prespecified adjustment covariates. With this specification, the main fixation-category coefficient represents the association between coding-defined fixation category and 90-day readmission at age 70 years. Marginal estimates were also calculated at selected ages of 60, 70, and 80 years.

For descriptive analyses of readmission diagnoses and procedures, unweighted counts and DISCWT-weighted proportions were calculated within each fixation cohort among patients who experienced 90-day readmission. The most frequent individual diagnosis and procedure codes, as well as grouped diagnosis and procedure categories, were reported as supplementary descriptive analyses to provide clinical context for the all-cause readmission endpoint. Statistical significance was defined as a 2-sided *p*-value < 0.05. Analyses were performed using SPSS (IBM Corp., Armonk, NY, USA).

### Ethics statement

The NRD is a publicly available, de-identified dataset; therefore, this study was exempt from institutional review board approval and informed consent requirements in accordance with U.S. federal regulations. All analyses complied with the HCUP Data Use Agreement.

## Results

### Study cohort

Before matching, the analytic cohort included 240,890 unweighted admissions, consisting of 217,750 coding-defined cemented-category and 23,140 coding-defined cementless-category primary TKA admissions. After 1:1 propensity-score matching, the matched analytic cohort included 23,140 unweighted admissions in each coding-defined fixation category. After applying DISCWT, these corresponded to weighted national estimates of 40,490 cemented-category and 40,501 cementless-category primary TKA admissions.

Baseline demographic, clinical, hospital-level, socioeconomic, and procedural characteristics were well balanced between coding-defined fixation categories after propensity-score matching (Table 1). Before matching, imbalance was observed for several covariates, most notably robotic-assisted surgery. After 1:1 matching, all available propensity-score covariates demonstrated acceptable balance, with all absolute post-matching standardized mean differences below 0.10. The maximum absolute pre-match SMD was 0.601 in the unweighted sample and 0.573 after applying DISCWT, whereas the maximum absolute post-match SMD was 0.061 in the unweighted matched sample and 0.059 after applying DISCWT. Full pre- and post-matching balance diagnostics are provided in Supplementary Table S1.

**Table 1:** Baseline characteristics of the propensity-matched cohort

Characteristic	Cemented-category weighted *n* = 40,490	Cementless-category weighted *n* = 40,501	*p* value
Age, years, mean ± SD	65.24 ± 9.56	65.34 ± 9.57	0.15
Female, %	52.8	53.2	0.24
Hypertension, %	57.8	57.6	0.50
Type 2 diabetes mellitus, %	21.9	22.0	0.61
Obstructive sleep apnea, %	17.8	17.3	0.07
Chronic anemia, %	5.3	5.5	0.28
Osteoporosis, %	2.7	2.9	0.09
Obesity, %	38.3	38.7	0.33
Liver disease, %	1.9	1.9	0.98
Thyroid disorders, %	16.1	16.3	0.50
Robotic-assisted surgery, %	29.1	29.1	0.98

Note: Values are shown for the propensity-score matched cohort. Propensity-score matching was performed on the unweighted analytic sample before application of DISCWT. The matched analytic cohort included 23,140 unweighted admissions in each coding-defined fixation category. DISCWT-weighted national estimates were 40,490 cemented-category and 40,501 cementless-category admissions. Full pre- and post-matching standardized mean differences for all propensity-score covariates are provided in Supplementary Table S1

### Index hospitalization outcomes

During the index hospitalization, most coded major complications occurred at low rates. Blood transfusion rates were comparable between the coding-defined cemented-category and cementless-category cohorts (1.0% vs 1.1%; OR 1.09, 95% CI 0.95–1.25; *p* = 0.203), despite a lower coded rate of blood loss anemia in the cementless-category cohort (Table 2). Because these outcomes were identified using administrative diagnosis and procedure codes, they should be interpreted as coded early complications rather than adjudicated clinical events.

**Table 2:** Key clinical outcomes in the propensity-matched cohort

Outcome	Cemented-category	Cementless-category	OR (95% CI)	*p* value
90-day readmission, %	5.8	5.2	0.89 (0.84–0.95)	< 0.001
Intraoperative fracture, %	0.2	0.3	1.67 (1.25–2.23)	0.001
Blood loss anemia, %	13.1	11.3	0.84 (0.81–0.88)	< 0.001
Blood transfusion, %	1.0	1.1	1.09 (0.95–1.25)	0.203
Any in-hospital complication, %	15.4	13.5	0.86 (0.83–0.90)	< 0.001

Two clinically relevant differences were observed. Intraoperative fracture occurred more frequently in the cementless-category group (0.3% vs 0.2%) and remained independently associated with coding-defined cementless-category fixation (OR 1.67, 95% CI 1.25–2.23; *p* = 0.001) (Table 2).

In contrast, coded blood loss anemia was less frequent in the cementless-category group (11.3% vs 13.1%) and was independently associated with lower odds in adjusted analysis (OR 0.84, 95% CI 0.81–0.88; *p* < 0.001) (Table 2), whereas blood transfusion rates were similar between groups.

A composite endpoint of any in-hospital complication occurred in 15.4% of cemented cases and 13.5% of cementless cases (*p* < 0.001). In adjusted analysis, coding-defined cementless-category fixation remained associated with lower odds of experiencing any complication (OR 0.86, 95% CI 0.83–0.90; *p* < 0.001) (Table 2).

### Ninety-day readmission

In the matched cohort, the weighted 90-day readmission proportion was 5.76% in the cemented-category group and 5.17% in the cementless-category group. On adjusted logistic regression, coding-defined cementless-category TKA was associated with lower odds of 90-day readmission compared with cemented-category TKA (OR 0.89, 95% CI 0.84–0.95; *p* < 0.001) (Table 2). Among patients who underwent readmission, time to readmission did not differ significantly between groups (28.67 ± 24.88 vs 28.41 ± 24.58 days, *p* = 0.73). However, readmissions following cemented-category TKA were associated with longer length of stay (6.22 ± 8.81 vs 4.63 ± 4.69 days, *p* < 0.001) and higher total charges ($74,985.69 ± $139,172.69 vs $61,072.91 ± $72,770.30, *p* < 0.001) (Table 3). Because readmission diagnoses and procedures were heterogeneous, these readmission-burden findings should be interpreted as inpatient administrative resource-utilization measures rather than direct evidence of fixation-related failure.

**Table 3:** Readmission burden among patients with 90-day readmission

Readmission measure	Cemented-category	Cementless-category	*p* value
Days to readmission, mean ± SD	28.67 ± 24.88	28.41 ± 24.58	0.726
Readmission length of stay, days, mean ± SD	6.22 ± 8.81	4.63 ± 4.69	< 0.001
Readmission total charges, USD, mean ± SD	74,985.69 ± 139,172.69	61,072.91 ± 72,770.30	< 0.001

### Primary causes and procedures during 90-day readmission

Among patients who experienced 90-day readmission, grouped primary readmission diagnoses were heterogeneous in both coding-defined fixation categories and included prosthesis-related infection, wound complications, cellulitis or soft-tissue infection, sepsis or bacteremia, thromboembolic events, cardiopulmonary diagnoses, gastrointestinal diagnoses, renal or urinary diagnoses, mechanical or fracture-related diagnoses, and other or mixed medical categories (Supplementary Table S2). Among cemented-category readmissions, the most common grouped diagnosis categories were other or mixed diagnoses, prosthesis-related infection, cellulitis or soft-tissue infection, cardiopulmonary diagnoses, and sepsis or bacteremia. Among cementless-category readmissions, the most common grouped diagnosis categories were other or mixed diagnoses, prosthesis-related infection, gastrointestinal diagnoses, cardiopulmonary diagnoses, and sepsis or bacteremia. Prosthesis-related infection was the primary readmission diagnosis in 188 unweighted readmissions in the cemented-category group and 165 unweighted readmissions in the cementless-category group, corresponding to 15.87% and 13.64% of DISCWT-weighted readmissions, respectively. When expressed using the overall matched cohort denominator, prosthesis-related infection readmission occurred in 0.81% of unweighted cemented-category admissions and 0.71% of unweighted cementless-category admissions. Using DISCWT-weighted counts and the overall matched cohort denominator, the corresponding cohort-denominator proportions were 0.91% and 0.71%, respectively. These values should be interpreted as primary-diagnosis prosthesis-related infection readmissions within 90 days and not as total infection rates, outpatient infection events, or implant-level failure rates. These descriptive findings indicate that 90-day readmissions were not attributable to a single fixation-specific failure mechanism.

Grouped principal procedures during readmission were also heterogeneous and were not limited to knee revision-related procedures (Supplementary Table S3). A substantial proportion of readmissions had no principal procedure coded. Knee revision, removal, or replacement-related procedures accounted for a minority of readmissions in both coding-defined fixation categories, while other procedure groups included wound, soft-tissue, or tendon procedures; non-knee orthopedic procedures; cardiopulmonary, vascular, or access procedures; gastrointestinal, hepatobiliary, or abdominal procedures; renal, urinary, or urogenital procedures; administration, transfusion, or medication-related procedures; rehabilitation or substance-related procedures; and other non-knee procedures. These descriptive findings further support cautious interpretation of all-cause readmission as an administrative endpoint rather than a direct surrogate for fixation failure, implant performance, or component-level survivorship.

### Interaction between fixation type and age

In the revised age-centered interaction model, age was centered at 70 years. Therefore, the main fixation-category coefficient represents the association between coding-defined cementless-category versus cemented-category TKA and 90-day readmission at age 70 years. At age 70 years, coding-defined cementless-category TKA was not significantly associated with 90-day readmission compared with cemented-category TKA (OR 0.96, 95% CI 0.90–1.02; *p* = 0.189). The fixation-category × age interaction was statistically significant, corresponding to an OR of 1.019 per additional year of age (95% CI 1.012–1.026; *p* < 0.001). Marginal estimates suggested an OR of 0.79 at age 60 years, 0.96 at age 70 years, and 1.16 at age 80 years. These findings should be interpreted cautiously as exploratory short-term administrative associations rather than evidence of implant performance or durability (Table 4; Supplementary Table S4).

**Table 4:** Readmission burden among patients with 90-day readmission

Variable	Adjusted OR	95% CI	*p* value
Cementless-category fixation at age 70 years	0.96	0.90–1.02	0.189
Age, per additional year from age 70	1.012	1.007–1.016	< 0.001
Cementless-category × age interaction	1.019	1.012–1.026	< 0.001
Female sex	0.886	0.834–0.942	< 0.001

Note: Age was centered at 70 years. Therefore, the fixation-category estimate represents the adjusted association between coding-defined cementless-category and cemented-category TKA at age 70 years. Marginal estimates at ages 60, 70, and 80 years are provided in Supplementary Table S4

Additional descriptive details on the leading individual primary diagnosis codes and leading individual principal procedure codes during 90-day readmission are provided in Supplementary Tables S5 and S6, respectively.

## Discussion

In this propensity score-matched nationwide administrative analysis of elective unilateral primary TKA performed for osteoarthritis, ICD-10-PCS coding-defined cementless-category and cemented-category admissions demonstrated modest differences in selected early administrative outcomes. Cementless-category TKA was associated with a slightly lower weighted 90-day readmission proportion, lower coded rates of blood loss anemia and overall in-hospital complications, and a higher coded rate of intraoperative fracture. In the revised age-centered interaction analysis, the fixation-category association at age 70 years was not statistically significant, although the fixation-category by age interaction remained significant. Taken together, these findings suggest that coding-defined fixation categories were associated with different short-term perioperative administrative risk profiles, not evidence of implant superiority, radiographic fixation, osseointegration, durability, or long-term survivorship benefit.

One clinically relevant finding was the tradeoff between lower coded perioperative morbidity and higher coded intraoperative fracture risk. Coding-defined cementless-category TKA was associated with lower odds of coded blood loss anemia and a lower composite coded complication rate. However, blood transfusion rates were comparable between cohorts, suggesting that the observed difference in blood loss anemia may not necessarily represent a clinically meaningful difference in severe perioperative bleeding. Instead, this discrepancy may reflect coding practices, differences in documentation thresholds, perioperative hemoglobin monitoring, or administrative classification of postoperative anemia. Therefore, the blood loss anemia finding should be interpreted cautiously, particularly because transfusion rates were similar between groups.

At the same time, the increased coded odds of intraoperative fracture in the cementless-category group are clinically relevant and biologically plausible, given that cementless implantation often relies on press-fit stability and may place greater stress on bone during component insertion. Although the absolute fracture rate remained low, this signal should be interpreted within the limitations of administrative coding. The NRD does not provide operative reports, implant-specific information, bone quality, surgeon technique, fracture morphology, treatment details, or clinical adjudication of the coded fracture event [6, 12–14].

The difference in 90-day readmission between coding-defined fixation categories was statistically significant but modest in absolute terms. This distinction is important because, in large administrative datasets, small differences may reach statistical significance while their clinical meaning depends on the underlying causes of readmission. In the present analysis, grouped primary readmission diagnoses were heterogeneous and included prosthesis-related infection, wound complications, cellulitis or soft-tissue infection, sepsis or bacteremia, thromboembolic events, cardiopulmonary diagnoses, gastrointestinal diagnoses, renal or urinary diagnoses, mechanical or fracture-related diagnoses, and other or mixed medical categories. Therefore, the observed readmission difference should not be interpreted as a direct surrogate for fixation-related failure, implant performance, or component-level survivorship. Rather, these findings suggest that coding-defined fixation categories were associated with different short-term administrative readmission patterns within the limitations of an administrative readmission database.

Analysis of grouped principal procedures during readmission provided additional context. Although knee revision, removal, or replacement-related procedure codes were identified in both coding-defined fixation categories, these represented only a minority of readmissions. A substantial proportion of readmissions had no principal procedure coded, and many coded procedures reflected broader inpatient care, including wound, soft-tissue, or tendon procedures; non-knee orthopedic procedures; cardiopulmonary, vascular, or access procedures; gastrointestinal, hepatobiliary, or abdominal procedures; renal, urinary, or urogenital procedures; administration, transfusion, or medication-related procedures; rehabilitation or substance-related procedures; and other non-knee procedures. These findings emphasize that 90-day readmission following TKA is a mixed administrative endpoint that captures both orthopaedic and non-orthopaedic events. Accordingly, procedure-code findings should be considered descriptive and exploratory, not definitive measures of fixation failure, revision burden, implant performance, or component-level survivorship.

The revised age-interaction analysis centered age at 70 years, allowing the main fixation-category estimate to be interpreted at a clinically meaningful reference age. At age 70 years, coding-defined cementless-category TKA was not significantly associated with 90-day readmission compared with cemented-category TKA. The statistically significant fixation-category by age interaction suggests that the association between coding-defined fixation category and readmission differed across age. Marginal estimates suggested lower readmission odds for the cementless-category group at age 60 years, no significant association at age 70 years, and higher readmission odds at age 80 years. These findings should be interpreted cautiously as exploratory administrative associations and may reflect age-related differences in patient selection, perioperative risk, coding patterns, bone quality, surgeon preference, implant selection, or unmeasured confounding rather than a causal fixation effect [15–17].

Among readmitted patients, cemented TKA was associated with longer readmission length of stay and higher readmission hospital charges, despite similar time to readmission. These findings suggest that readmission burden differed between cohorts among patients who required rehospitalization. However, because readmission diagnoses and procedures were heterogeneous and the NRD does not provide full clinical context, operative reports, outpatient costs, post-discharge rehabilitation costs, or longitudinal episode-of-care expenditures, these charge and length-of-stay findings should be interpreted as markers of inpatient resource utilization rather than comprehensive cost or value comparisons between fixation strategies [18, 19].

Our findings should be interpreted in the context of prior literature. Previous comparisons of cemented and cementless TKA have produced mixed results, partly due to differences in patient selection, implant generations, surgeon experience, and study design. Registry studies and institutional cohorts often provide long-term survivorship data but may be limited in their ability to capture short-term readmission burden and resource utilization at a national level [20]. Conversely, administrative database studies offer broad contemporary representation but are dependent on coding accuracy and lack granular implant-specific and radiographic data. The present study contributes to this literature by focusing on a recent national cohort, restricting the analysis to a more homogeneous osteoarthritis population, excluding contralateral arthroplasty readmissions, and applying propensity score matching to reduce measured baseline imbalance.

This study has several limitations. First, as with all retrospective administrative database studies, the analysis is subject to residual confounding despite propensity score matching and multivariable adjustment. Although the propensity model incorporated available demographic, socioeconomic, hospital-level, procedural, and comorbidity variables, the NRD does not capture all clinically relevant factors that influence fixation selection and postoperative risk, including bone quality, frailty, activity level, body habitus beyond coded obesity, severity of deformity, neurologic conditions not consistently coded, surgeon preference, institutional protocols, implant design, cementing technique, and intraoperative decision-making. Therefore, unmeasured selection bias remains possible.

Second, fixation status was defined using ICD-10-PCS administrative procedure codes. These codes do not provide implant-level details, manufacturer information, cementing technique, component-level fixation status, operative reports, radiographic findings, or confirmation of whether a construct was fully cemented, fully cementless, or hybrid. As a result, the exposure should be interpreted strictly as coding-defined cemented-category versus cementless-category primary TKA rather than definitive component-level fixation classification. The NRD also cannot assess osseointegration, radiographic fixation, implant migration, durability, or long-term survivorship.

Third, the NRD is limited to inpatient administrative data and does not capture outpatient complications, emergency department visits without admission, rehabilitation events, outpatient antibiotic treatment, nonoperative wound management, patient-reported outcome measures, functional recovery, radiographic alignment, radiographic loosening, osseointegration, implant migration, or long-term survivorship. Consequently, the present findings should not be interpreted as evidence of implant superiority, radiographic durability, or long-term fixation success.

Fourth, complications, readmission diagnoses, and procedures were identified using ICD-10-CM and ICD-10-PCS codes and are therefore subject to coding error, documentation variability, and misclassification. Blood loss anemia, in particular, may reflect coding or documentation thresholds rather than clinically significant bleeding, especially because transfusion rates were comparable between cohorts. Similarly, procedure codes during readmission could not be verified against operative reports, laterality, indication, component-level revision details, or relationship to the index knee arthroplasty. Procedure-related findings should therefore be interpreted as descriptive and exploratory.

Fifth, although the NRD enables tracking of readmissions within a calendar year, it cannot follow patients across calendar years or across all potential care settings. Index admissions after September were excluded to allow a complete 90-day follow-up window, but readmissions outside participating states or outside the inpatient setting may still be missed. Finally, hospital charges reflect billed inpatient charges rather than actual costs, reimbursements, outpatient expenditures, rehabilitation costs, revision costs after 90 days, or total episode-of-care costs.

Despite these limitations, the study has notable strengths. It evaluates a large, contemporary, nationally representative cohort; uses a focused primary TKA population limited to osteoarthritis and day-0 surgery; applies 1:1 propensity score matching; and examines both perioperative complications and 90-day readmission outcomes. These features enhance the clinical relevance of the findings and provide a practical perspective on how fixation strategy may influence early postoperative outcomes in current U.S. practice.

## Conclusion

In this U.S. nationwide propensity-matched administrative analysis of elective unilateral primary TKA for osteoarthritis, ICD-10-PCS coding-defined cementless-category and cemented-category admissions demonstrated modest differences in selected early complications and 90-day readmission patterns. The 1:1 matched analytic cohort included equal unweighted group sizes, while DISCWT-weighted national estimates were reported separately. Coding-defined cementless-category TKA was associated with a slightly lower weighted 90-day readmission proportion and lower weighted proportions of selected coded early complications, but with a higher weighted proportion of intraoperative fracture. Blood transfusion rates were comparable despite lower coded blood loss anemia in the cementless-category cohort. Among readmitted patients, grouped readmission diagnoses and procedures were heterogeneous and were not limited to fixation-related or knee revision-related events. In the revised age-centered interaction analysis, the fixation-category association at age 70 years was not statistically significant, although the fixation-category by age interaction remained significant. These findings should be interpreted as short-term administrative associations only and should not be considered evidence of implant superiority, fixation failure, radiographic fixation, osseointegration, durability, radiographic survivorship, or long-term clinical benefit.

## Supplementary Information

Supplementary Material 1.

## Data Availability

This study used publicly available data from the HCUP Nationwide Readmissions Database (NRD). Data can be obtained directly from HCUP (https://hcup-us.ahrq.gov/databases.jsp) upon purchase.
