# Supplementary material for: Ninety-day readmission and early complications after coding-defined cementless-category versus cemented-category primary total knee arthroplasty
Source: Arthroplasty. 2026 Aug 5;8:57. doi: 10.1186/s42836-026-00418-2 (PMC13439900; doi:10.1186/s42836-026-00418-2)
Supplement: Supplementary file 1 [file 42836_2026_418_MOESM1_ESM.docx]

**Supplementary Material**

Ninety-day readmission and early complications after coding-defined cementless-category versus cemented-category primary total knee arthroplasty

This supplementary material includes the ICD-10-PCS code-to-category mapping used to define the coding-defined fixation cohorts, complete propensity-score balance diagnostics, grouped readmission diagnosis and procedure summaries, age-centered interaction analyses, and leading individual readmission diagnosis and procedure codes. Counts are unweighted unless otherwise specified; percentages are DISCWT-weighted proportions within the relevant coding-defined fixation category.

**Supplementary Table S1.** Covariate balance before and after propensity-score matching

| **Covariate** | **Level** | **Before matching SMDunweighted** | **Before matching SMDDISCWT-weighted** | **After matching SMDunweighted** | **After matching SMDDISCWT-weighted** |
| --- | --- | --- | --- | --- | --- |
| Age, years | Continuous | −0.298 | −0.302 | 0.002 | 0.010 |
| Female sex | Yes vs no | −0.205 | −0.199 | −0.000 | 0.008 |
| Primary expected payer | Medicare | −0.229 | −0.233 | −0.046 | −0.037 |
| Primary expected payer | Medicaid | 0.039 | 0.041 | −0.040 | −0.050 |
| Primary expected payer | Private insurance | 0.189 | 0.194 | 0.061 | 0.059 |
| Primary expected payer | Self-pay | −0.008 | −0.003 | −0.008 | −0.002 |
| Primary expected payer | No charge | −0.013 | −0.013 | −0.046 | −0.057 |
| Primary expected payer | Other | 0.077 | 0.072 | 0.021 | 0.017 |
| Median household income quartile | Quartile 1 (lowest) | −0.007 | 0.006 | −0.037 | −0.033 |
| Median household income quartile | Quartile 2 | −0.011 | −0.014 | 0.035 | 0.036 |
| Median household income quartile | Quartile 3 | −0.019 | −0.031 | −0.025 | −0.035 |
| Median household income quartile | Quartile 4 (highest) | 0.038 | 0.041 | 0.025 | 0.032 |
| Index year | 2020 | −0.130 | −0.125 | −0.002 | 0.003 |
| Index year | 2021 | 0.070 | 0.064 | 0.002 | −0.011 |
| Index year | 2022 | 0.074 | 0.076 | 0.000 | 0.008 |
| Robotic-assisted surgery | Yes vs no | 0.601 | 0.573 | 0.003 | −0.018 |
| Navigation-guided surgery | Yes vs no | 0.030 | 0.065 | 0.001 | 0.011 |
| Hypertension | Yes vs no | −0.005 | −0.001 | −0.011 | −0.005 |
| Dyslipidemia | Yes vs no | −0.023 | −0.025 | 0.003 | 0.009 |
| Sleep apnea | Yes vs no | 0.040 | 0.034 | −0.005 | −0.013 |
| Chronic anemia | Yes vs no | −0.022 | −0.027 | 0.016 | 0.008 |
| Smoking | Yes vs no | 0.019 | 0.017 | −0.005 | −0.014 |
| Drug abuse | No variation | 0.000 | 0.000 | 0.000 | 0.000 |
| Alcohol abuse | Yes vs no | 0.007 | 0.006 | 0.029 | 0.020 |
| Osteoporosis | Yes vs no | −0.108 | −0.103 | 0.010 | 0.012 |
| Mental health disorder | Yes vs no | 0.036 | 0.036 | −0.015 | −0.015 |
| Parkinson disease | Yes vs no | −0.012 | −0.008 | −0.059 | −0.047 |
| Alzheimer disease | Yes vs no | −0.019 | −0.019 | −0.017 | −0.013 |
| Type 2 diabetes | Yes vs no | −0.023 | −0.018 | 0.005 | 0.004 |
| Diabetes mellitus | Yes vs no | −0.023 | −0.018 | 0.004 | −0.002 |
| Renal disease | Yes vs no | −0.072 | −0.074 | 0.028 | 0.034 |
| Congestive heart failure | Yes vs no | −0.031 | −0.031 | 0.032 | 0.032 |
| Chronic lung disease | Yes vs no | −0.045 | −0.049 | −0.016 | −0.017 |
| Liver disease | Yes vs no | 0.002 | 0.002 | −0.003 | 0.000 |
| Thyroid disorder | Yes vs no | −0.059 | −0.058 | −0.005 | 0.005 |
| Obesity | Yes vs no | 0.066 | 0.066 | 0.005 | 0.007 |

***Note:*** SMD indicates standardized mean difference. Absolute SMD values below 0.10 were considered acceptable. Matching was performed on the unweighted analytic sample; DISCWT-weighted SMDs are shown for transparency.

**Supplementary Table S2.** Grouped principal diagnoses during 90-day readmission

| **Grouped principal readmission diagnosis** | **Cemented unweighted, n** | **Cemented weighted, n** | **Cemented weighted, %** | **Cementless unweighted, n** | **Cementless weighted, n** | **Cementless weighted, %** |
| --- | --- | --- | --- | --- | --- | --- |
| Prosthesis-related infection | 188 | 370.1 | 15.87 | 165 | 285.7 | 13.64 |
| Wound complications | 31 | 41.8 | 1.79 | 52 | 101.4 | 4.84 |
| Cellulitis / soft-tissue infection | 101 | 275.1 | 11.80 | 29 | 53.4 | 2.55 |
| Sepsis/bacteremia | 73 | 136.3 | 5.84 | 81 | 140.5 | 6.71 |
| Thromboembolic | 50 | 106.1 | 4.55 | 46 | 77.0 | 3.67 |
| Cardiopulmonary | 157 | 246.4 | 10.57 | 125 | 226.7 | 10.82 |
| Gastrointestinal | 71 | 120.0 | 5.14 | 148 | 263.0 | 12.55 |
| Renal/urinary | 42 | 101.1 | 4.34 | 42 | 73.6 | 3.51 |
| Mechanical / fracture-related | 48 | 78.1 | 3.35 | 39 | 72.4 | 3.46 |
| Neurologic/cerebrovascular | 34 | 64.0 | 2.74 | 22 | 41.8 | 1.99 |
| Postoperative pain/hematoma | 20 | 41.7 | 1.79 | 17 | 28.8 | 1.37 |
| Anemia / blood-loss | 4 | 7.2 | 0.31 | 10 | 16.0 | 0.76 |
| Syncope/hypotension | 15 | 24.5 | 1.05 | 9 | 15.4 | 0.73 |
| Other/mixed | 419 | 720.2 | 30.87 | 384 | 699.7 | 33.39 |

**Note:** Counts are unweighted readmission counts. Percentages are DISCWT-weighted proportions among 90-day readmissions within each coding-defined fixation category. Categories are descriptive and do not establish implant-level mechanisms. Prosthesis-related infection values in this table are proportions among readmitted patients; corresponding overall matched cohort-denominator rates are reported in the main Results text.

**Supplementary Table S3.** Grouped principal procedures during 90-day readmission

| **Grouped principal procedure during readmission** | **Cemented unweighted, n** | **Cemented weighted, n** | **Cemented weighted, %** | **Cementless unweighted, n** | **Cementless weighted, n** | **Cementless weighted, %** |
| --- | --- | --- | --- | --- | --- | --- |
| No principal procedure coded | 439 | 781.8 | 33.52 | 489 | 872.8 | 41.66 |
| Knee revision/removal/replacement-related | 139 | 241.1 | 10.33 | 118 | 215.1 | 10.27 |
| Other knee joint procedure | 64 | 119.1 | 5.11 | 58 | 106.4 | 5.08 |
| Wound / soft-tissue / tendon procedure | 125 | 259.3 | 11.12 | 83 | 154.7 | 7.38 |
| Non-knee orthopedic procedure | 95 | 159.2 | 6.82 | 79 | 153.7 | 7.33 |
| Cardiopulmonary/vascular / access procedure | 125 | 199.5 | 8.55 | 115 | 201.2 | 9.60 |
| Gastrointestinal/hepatobiliary/abdominal procedure | 101 | 248.7 | 10.66 | 114 | 197.9 | 9.45 |
| Renal/urinary/urogenital procedure | 30 | 64.4 | 2.76 | 20 | 34.7 | 1.66 |
| Administration/transfusion/medication | 92 | 162.4 | 6.96 | 34 | 60.2 | 2.87 |
| Rehabilitation / substance-related procedure | 21 | 60.9 | 2.61 | 5 | 11.5 | 0.55 |
| Other non-knee procedure | 22 | 36.2 | 1.55 | 54 | 87.2 | 4.16 |

***Note:*** Counts are unweighted readmission counts. Percentages are DISCWT-weighted proportions among 90-day readmissions within each coding-defined fixation category. Categories are descriptive and include non-knee procedures.

**Supplementary Table S4A.** Age-centered fixation-category interaction for 90-day readmission

| **Estimate** | **OR** | **95% CI** | ***p* value** |
| --- | --- | --- | --- |
| Cementless-category effect at age 70 years | 0.957 | 0.897–1.022 | 0.189 |
| Fixation × age interaction per 1-year increase | 1.019 | 1.012–1.026 | <0.001 |
| Cementless-category vs cemented-category at age 60 years | 0.793 | 0.733–0.859 | <0.001 |
| Cementless-category vs cemented-category at age 70 years | 0.957 | 0.897–1.022 | 0.189 |

**Supplementary Table S4B.** Age-centered marginal estimates at selected ages

| **Age, years** | **Category/contrast** | **Adjusted predicted readmission, %** | **95% CI, %** |
| --- | --- | --- | --- |
| 60 | Cemented-category | 5.550 | 5.311–5.854 |
| 60 | Cementless-category | 4.473 | 4.210–4.738 |
| 60 | Risk difference: cementless − cemented | −1.077 | −1.466–0.778 |
| 70 | Cemented-category | 5.827 | 5.570–6.145 |
| 70 | Cementless-category | 5.598 | 5.346–5.827 |
| 70 | Risk difference: cementless − cemented | −0.230 | −0.606–0.096 |
| 80 | Cemented-category | 6.118 | 5.605–6.735 |
| 80 | Cementless-category | 6.979 | 6.425–7.544 |

***Note:*** Age was centered at 70 years. The main fixation-category estimate is therefore interpretable at age 70 years. Marginal estimates are descriptive adjusted predictions from the age-centered interaction model.

**Supplementary Table S5.** Leading individual primary diagnoses during 90-day readmission by fixation cohort

| **Cohort** | **Rank** | **ICD-10-CM code** | **Primary diagnosis category** | **n** | **Weighted, %** |
| --- | --- | --- | --- | --- | --- |
| Cemented | 1 | L03116 | Cellulitis of left lower limb | 61 | 9.3 |
| Cemented | 2 | T8453XA | Infection/inflammatory reaction due to internal right knee prosthesis | 72 | 5.4 |
| Cemented | 3 | T8143XA | Postprocedural/surgical-site infection | 31 | 4.2 |
| Cemented | 4 | T8454XA | Infection/inflammatory reaction due to internal left knee prosthesis | 56 | 4.1 |
| Cemented | 5 | A419 | Sepsis, unspecified organism | 53 | 4.1 |
| Cementless | 1 | T8454XA | Infection/inflammatory reaction due to internal left knee prosthesis | 66 | 5.3 |
| Cementless | 2 | T8453XA | Infection/inflammatory reaction due to internal right knee prosthesis | 61 | 5.1 |
| Cementless | 3 | A419 | Sepsis, unspecified organism | 61 | 4.9 |
| Cementless | 4 | T8131XA | Wound disruption/dehiscence | 36 | 3.5 |
| Cementless | 5 | I110 | Hypertensive heart disease with heart failure | 17 | 1.7 |

***Note:*** Values are reported among patients who experienced 90-day readmission. Counts are unweighted. Percentages represent DISCWT-weighted proportions within each coding-defined fixation category among readmitted patients. This table reports leading individual ICD-10-CM diagnosis codes and complements the grouped diagnosis summary in Supplementary Table S2.

**Supplementary Table S6.** Leading individual principal procedures during 90-day readmission by fixation cohort

| **Cohort** | **Rank** | **ICD-10-PCS code** | **Principal procedure category** | **n** | **Weighted, %** |
| --- | --- | --- | --- | --- | --- |
| Cemented | 1 | 0DB98ZX | Endoscopic gastrointestinal biopsy / diagnostic excision | 37 | 6.1 |
| Cemented | 2 | 0JBN0ZZ | Lower-limb soft-tissue debridement/excision | 29 | 4.2 |
| Cemented | 3 | 30233N1 | Blood transfusion | 51 | 4.0 |
| Cemented | 4 | 0SPC09Z | Knee implant removal or revision-related procedure | 43 | 3.1 |
| Cemented | 5 | HZ2ZZZZ | Detoxification or substance-use treatment | 18 | 2.5 |
| Cementless | 1 | 0SPC09Z | Knee implant removal or revision-related procedure | 26 | 2.4 |
| Cementless | 2 | 0SPD09Z | Knee implant removal or revision-related procedure | 25 | 2.4 |
| Cementless | 3 | 0SPC0JZ | Knee prosthesis/component removal or revision-related procedure | 20 | 1.6 |
| Cementless | 4 | 30233N1 | Blood transfusion | 16 | 1.3 |
| Cementless | 5 | 0SPD0JZ | Knee prosthesis/component removal or revision-related procedure | 11 | 1.0 |

***Note:*** Values are reported among patients who experienced 90-day readmission. Counts are unweighted. Percentages represent DISCWT-weighted proportions within each coding-defined fixation category among readmitted patients. Blank or missing principal procedure codes were not included among the ranked individual procedures; however, no principal procedure was coded in 33.5% of weighted cemented-category readmissions and 41.7% of weighted cementless-category readmissions. This table reports leading individual ICD-10-PCS procedure codes and complements the grouped procedure summary in Supplementary Table S3. Procedure-code interpretation was descriptive and exploratory because the NRD does not provide operative reports, laterality confirmation relative to the index procedure, implant-level details, or component-level revision information.

**Supplementary Table S7.** ICD-10-PCS code-to-category mapping used to define coding-defined fixation cohorts

| **ICD-10-PCS code** | **Laterality** | **ICD-10-PCS description** | **Study category** |
| --- | --- | --- | --- |
| 0SRC0J9 | Right knee | Replacement of Right Knee Joint with Synthetic Substitute, Cemented, Open Approach | Coding-defined cemented-category primary TKA |
| 0SRD0J9 | Left knee | Replacement of Left Knee Joint with Synthetic Substitute, Cemented, Open Approach | Coding-defined cemented-category primary TKA |
| 0SRC0JA | Right knee | Replacement of Right Knee Joint with Synthetic Substitute, Uncemented, Open Approach | Coding-defined cementless-category primary TKA |
| 0SRD0JA | Left knee | Replacement of Left Knee Joint with Synthetic Substitute, Uncemented, Open Approach | Coding-defined cementless-category primary TKA |

***Note:*** Fixation category was assigned using the ICD-10-PCS qualifier in the primary procedure code. Codes with the cemented qualifier were assigned to the coding-defined cemented-category cohort, and codes with the uncemented qualifier were assigned to the coding-defined cementless-category cohort. Other primary knee arthroplasty codes were excluded if they lacked a cemented or uncemented qualifier, represented partial or compartment-specific knee arthroplasty constructs, involved non-synthetic or unspecified device categories, or could not be assigned reproducibly to one of the two mutually exclusive coding-defined fixation categories. These administrative categories should not be interpreted as implant-level confirmation of fully cemented, fully cementless, or hybrid component-level fixation.
